# Microstructural evidence of the toughening mechanisms of polyurethane reinforced with halloysite nanotubes under high strain-rate tensile loading

**DOI:** 10.1038/s41598-021-92663-5

**Published:** 2021-06-23

**Authors:** Rafaela Aguiar, Ronald E. Miller, Oren E. Petel

**Affiliations:** Department of Mechanical and Aerospace Engineering, Carleton University, Ottawa, ON K1S 5B6 Canada

**Keywords:** Polymers, Mechanical properties

## Abstract

In this study, we have investigated the relationship between the spherulitic morphology and the dynamic tensile response of polyurethane reinforced with Halloysite nanotubes (HNTs). The polyurethane prepolymer is partially silane end-capped and filled with only 0.8 wt.% of acid-treated Halloysite nanotubes. The resultant nanocomposite material presents a 35% higher spall strength compared to the neat polyurethane and 21% higher fracture toughness. We show evidence that the HNTs are not the toughening phase in the nanocomposite, but rather it is their influence on the resultant spherulitic structures which alters the polymer microstructure and leads to a tougher dynamic response. Microstructural characterization is performed via Scanning Electron Microscopy, Atomic Force Microscopy and Field Emission Scanning Electron Microscopy, and crystallinity examination via X-ray diffraction. The spherulitic structures present a brittle fracture character, while the interspherulitic regions are more ductile and show large deformation. The nanocomposite presents a finer and more rigid spherulitic structure, and a more energy dissipative fracture mechanism characterized by a rougher fracture surface with highly deformed interspherulitic regions.

## Introduction

Polyurethane (PU) based materials can be optimized for a wide range of stress and strain rate conditions, as they can be synthesized with different compositions and macromolecular structural organization. In recent years, PU formulations have been developed for new applications to increase the survivability of structures under high-strain-rate conditions, including against blast and ballistic loading events^1^. PU coatings or interlayers are currently implemented in transparent armor systems as a way to increase the survivability of high-strength ceramic layers and improve fragment containment. Transparent armor must protect against blast and ballistic threats while maintaining structural integrity and optical transparency. The high tensile-ductility and adhesive properties of PU aid in the containment of fragments from the strike-face and intermediate ceramic layers^2^. PU interlayers can also be used in toughening strategies, whereby the selective positioning of the polymeric interlayers in specific regions of composite laminates improves performance. This method provides an increase in the fracture toughness and impact properties without sacrificing in-plane mechanical properties^3^. However, so far it is not clear how the macromolecular structural features influence the failure mechanism of PU under dynamic loading conditions.

The PU domain-fragmented structure is composed of sequences of macrodiol and diisocyanate-chain extender segments, referred to as the soft and hard segments, respectively. The hard segments are glassy and/or crystalline with a glass transition temperature (Tg) above room temperature, and the soft segments are rubbery with Tg below room temperature^4^. The phase separation starts during the polymeric formation of the urethane or urea groupings, when the hard segments become incompatible with the soft segments. The phase separation depends strongly on the degree of hydrogen bonds formed between the urethane/urea linkages, the reaction conditions and the manufacturing process^5^. The hard segments can act as physical crosslink sites between the polymeric chains in thermoplastic PU based materials, and as they are stiffer than the soft segment matrix, they behave as a nanofiller strengthening phase within the PU’s macromolecular structure^6^. This microphase-separated structure tends to increase the overall ductility and deformation recoverability of thermoplastic PUs^7^.

The mechanical response of PU under dynamic conditions have been reported in the literature for different compositions and loading conditions. Sun et al.^8^ studied the molecular dependencies of the high-strain-rate impact response of PUs and polyureas using a laser-induced particle impact test under strain rate magnitudes between 10^6^ and 10^8^ s^−1^. Their results showed that more intense hydrogen bonding between hard and soft segments led to greater dynamic strengthening and stiffening. They observed substantial influence of both the soft segment molecular weight and the hydrogen bonding type on the impact response of the polymers. Sarva et al.^9^ studied the contributions of the PU microstructure on the rate-sensitivity of its material properties. The quasi-static response of the polymer was evaluated via uniaxial compression tests, and high-strain-rate compression tests were conducted using a split Hopkinson pressure bar with strain rates varying from 10^3^ and 10^4^ s^−1^. Their characterization indicated that an increase in phase mixing and hydrogen bonding between hard and soft segments, which was controlled by altering the soft segment molecular weight, resulted in increased strain rate sensitivity of the PU. Their PUs presented a considerable stiffening under high strain rate conditions. Chen et al.^10^ presented a methodology to predict the high strain rate behaviour of thermoplastic PU using a viscoelastic continuum damage model based on the Boltzmann superposition integral. The model parameters considered linear viscoelastic properties obtained from dynamic mechanical analysis experiments, and the model was calibrated using quasi-static compression experimental results. Their model was able to effectively predict the experimental behaviour of the PU under high strain rates, based on the comparison of data obtained from split-Hopkinson pressure bar experiments with model simulation results for strain rate up to 2000 s^−1^. Sain et al.^11^ developed a finite deformation rate dependent constitutive model to predict the mechanical response of PU and PU-Montmorillonite clay nanocomposites under different strain rates and strain amplitude conditions. The multiple relaxation times and viscous behaviour of the PU were found using frequency dependent tan δ data from dynamic mechanical analysis. The model predictions for uniaxial loading–unloading responses under different strain rates followed the same trends observed experimentally.

PUs can contain microdomains with different morphological architectures, and the occurrence of spherical, cylindrical or lamellar microdomains can be related to the hard segment content, chemical composition, thermal history, and synthesis condition^12^. The main crystalline morphological configuration present in polymers crystallized from melts are spherulites^13^. These structures in PUs consist of hard segment lamellar crystal bundles preferentially oriented in the radial direction of the spherulite, within a soft segment matrix^14,15^. The hard segment chains in the lamellar crystal bundle are aligned perpendicularly to the spherulite radius, as observed by Aneja and Wilkes^15^ in PUs prepared via a solution cast method using atomic force microscopy. Yanagihara et al.^7^ studied the structural behaviour of spherulites in melt-crystallized thermoplastic PU, the spherulite was stretched by tension to 200% elongation and released at 300 mm/s. Optical micrographs and small angle X-ray scattering results indicate that the spherulitic structure returned to the unstretched configuration after complete retraction. Their results indicate that the spherical shape recovery occurs due to the rubbery behaviour of the soft segment matrix in combination with the hard segment domains acting as physical crosslink anchoring sites. Pielichowska et al.^16^ studied PU/graphite nano-platelet composites with nano-filler content range from 0.3 to 4 wt.% in PU. The occurrence of smaller spherulites with higher crystallinity degree was found in composites reinforced with a low nano-platelet content of 0.5 and 1 wt.%. Their optical micrographs results indicated that the graphene acts as a nucleation agent during crystallization.

Halloysite nanotubes (HNTs) are tubular clay materials with an external surface composed of siloxane (Si–O–Si) groups and an internal surface consisting of a gibbsite octahedral structure of aluminol (Al–OH) groups. The HNTs are a polymorph of the kaolin group with characteristic dimensions of HNTs varying over ranges of 300–1500 nm in length, 40–120 nm for the outer diameter, and 15–100 nm for the inner diameter^17,18^. The reactivity of the HNTs is restricted to Al–OH and Si–OH groups exposed in defects of the HNT aluminosilicate layers and at the outer edges^19,20^. Zhou et al.^21^ reported the effect of HNTs on heterogeneous nucleation in PU. Their polarized optical microscopy images revealed a reduction in the spherulite size with incorporation of HNT varying from 0.5 to 2.0 wt.%. Li et al.^22^ investigated the role of HNT and organosilane-modified HNT as a nucleation agent in Poly (butylene adipate-co-terephthalate) (PBAT). Their polarized optical micrographs and differential scanning calorimetry (DSC) results demonstrated that an HNT content of approximately 2 wt.% in PBAT provoked a decrease in spherulite size and accelerated the crystallization of the polymer.

According to published studies, the introduction of a low concentration of HNT can considerably improve the quasi-static mechanical and thermal properties in PU based systems. Gong et al.^23^ synthesized nanocomposites based on uniformly dispersed HNTs in millable PU elastomer, their experimental results indicate an increase of 116% in tensile strength and 58% increase in elongation at break with the introduction of 1.0 wt.% of HNTs. Lin et al.^24^ synthesized self-healing and recyclable PU reinforced with HNT and reported a two-fold increase in tensile strength and three-fold increase in Young’s modulus for HNT’s content of 1 wt.%. Gaaz et al.^25^ produced nanocomposites based on thermoplastic PUs reinforced with phosphoric acid treated HNTs and untreated HNT. Comparing to the neat PU, the nanocomposites with a filler content of 2 wt.% presented an increase in tensile strength of 35% for the nanocomposite based on acid treated HNT, and 26% increase for the PU reinforced with untreated HNT. Gaaz et al.^25^ also proposed that the phosphoric acid treatment resulted in a better dispersion of the HNTs in the composites leading to superior mechanical properties. The acid treatment of HNT can lead to reactivity increase via a reaction involving the acid and both the inner and outer surfaces of the nanotubes. Therefore, the breakage of the HNT structures via dissolution of the AlO_6_ octahedral layers and the breakdown and collapse of SiO_4_ tetrahedral layers result in an increase number of potential sites for bonding^26^.

In our previous work^27^, we have described the synthesis process and characterization of HNT-PU nanocomposites based on PU prepolymer partially end-capped with a secondary aminoalkoxysilane, and an acid-treated HNT content of 0.8 wt.%. The partial prepolymer termination was performed in order to improve the interfacial compatibility between HNT and PU, and the selected weight fraction of HNT was low enough to maintain the transparency of the polymer as a thin film. Gas-gun spall testing results for a tensile strain rate magnitude of 10^4^ s^−1^ showed an increase of 35% in dynamic tensile (spall) strength and 21% fracture toughness for the nanocomposite compared to the neat polyurethane while maintaining transparency. The present work is a continuation of that study. Here, the microstructural fracture mechanism of the neat PU and HNT-PU nanocomposite are explained through: (i) morphological analysis of the spherulite structure via Field Emission Scanning Electron Microscope (FESEM); (ii) failure mechanism and fracture kinetics investigation via Scanning Electron Microscope (SEM) of the spalled plane; (iii) spall plane evolution analysis via cryogenic breakage of spalled samples and SEM micrographs; (iv) crystallinity analysis via X-ray diffraction (XRD); and (v) surface topology of the spherulite/interspherulitic interface using Atomic Force Microscopy (AFM).

Our characterizations show how the HNTs does not behave as a traditional toughening phase in a composite, but rather through its interaction with the PU’s hard domains it can interfere with the resultant spherulitic morphology. This gives rise to a tougher PU microstructure with a finer distribution of smaller and more rigid spherulites surrounded by ductile interspherulitic regions. Further, we illustrate how and why this finer microstructure exhibits a tougher dynamic response and improved properties.

## Results and discussions

**Figure 1:**
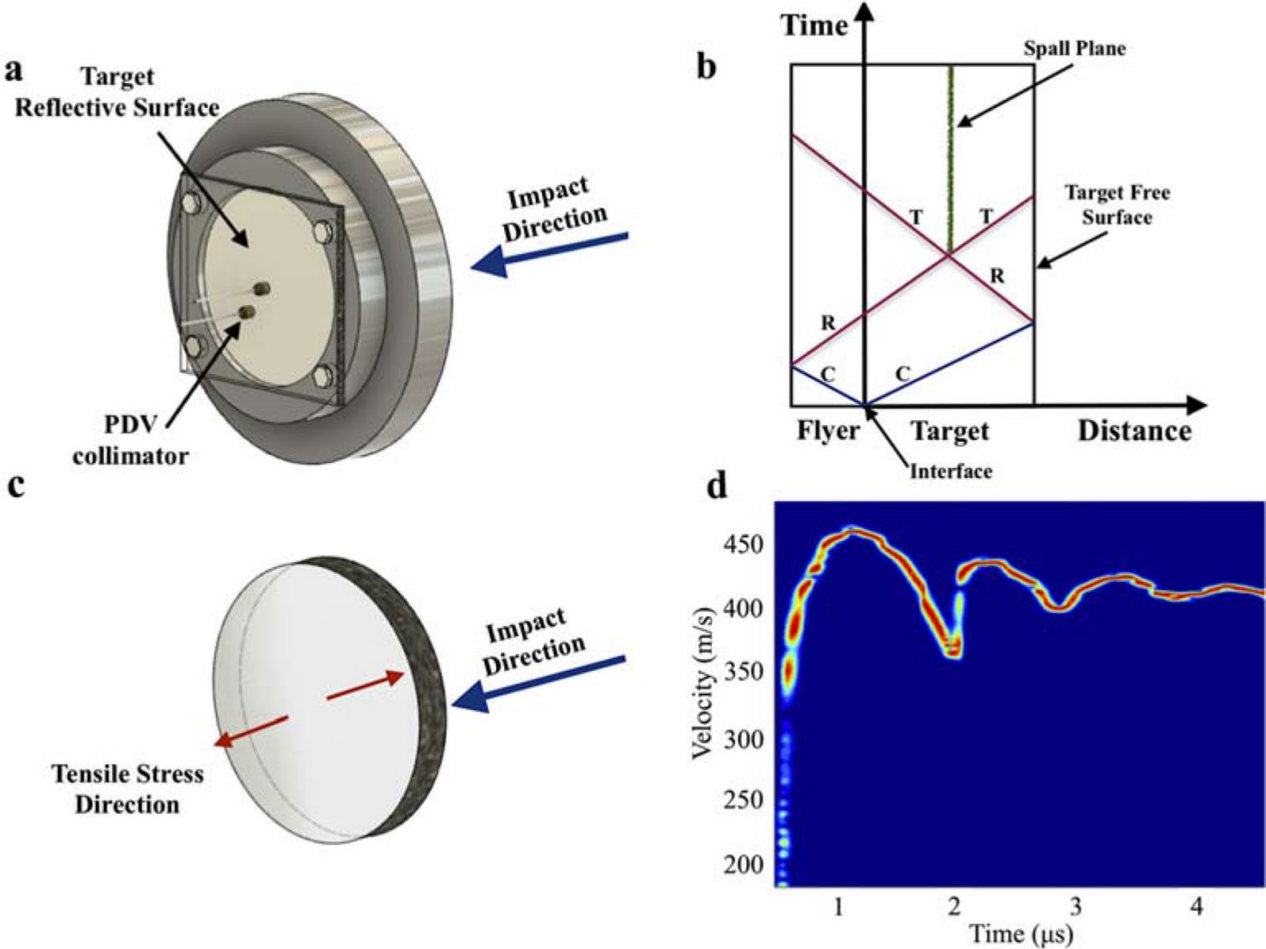
**(a)** Schematic configuration of the sample assembly used in the spall testing; **(b)** simplified distance-time plot showing the wave interaction in a plate impact configuration; **(c)** illustrative representation of the resultant tensile stress direction in the target **(d)** velocity history for HNT-PU nanocomposite.

The study of the microstructural fracture response was made through the analysis of recovered spalled samples, which were subjected to, and failed under, high-strain-rate tensile loads. Gas-gun spall experiments were conducted to measure the fracture toughness and spall strength of the polymeric samples. The schematic configuration of the target assembly can be seen on Fig. 1a. Spall testing provides a measure of the dynamic tensile strength of a specimen material. The geometry of an impactor and test specimen can be designed to generate a compression-expansion cycle that results in a planar dynamic tensile load within the specimen^28,29^. The wave dynamics that lead to this shock compression and subsequent isentropic expansion of the specimen material can be monitored through the motion of the rear free surface of the specimen (Fig. 1b).

Upon impact the test material achieves an equilibrium Hugoniot shock state at the peak velocity, after which the velocity decreases with the isentropic expansion of the material. The interaction of these expansion or rarefaction fans generates a tensile stress state within the target material. Figure 1b presents a simplified representation of the distance-time plot showing the wave propagations in a spall configuration, and the resultant tensile stress direction in the sample is illustrated on Fig. 1c. The spallation occurs if the tensile stress is high enough to initiate the process of nucleation growth, and coalescence of voids resulting in a failure plane (spall layer) within the target^30,31^, and the shape of the backface velocity history (Fig. 1d) allows one to determine the spall strength. The spalled samples were subjected to similar tensile strain rates (ε_u_) and comparable shock stresses. The samples presented spall strengths (σ_sp_) of 105 ± 2 MPa for the neat PU, 143 ± 3 MPa for the HNT-PU, and 129 ± 3 MPa for the nanocomposite without silane end-groups (HNT-PU*) for values of tensile strain rate during unloading varying from 2.75 to 2.79 (10^4^) s^−1^, and flyer velocities from 436 to 493 m/s. The fracture toughnesses (K_C_) were determined from spall tests that had similar values of shock stresses (0.96 GPa for the neat PU and 0.99 GPa for the nanocomposite). The fracture toughnesses were found to be 3.41 and 4.13 MPa m^1/2^ for the neat PU and nanocomposite, respectively^27^.

**Figure 2:**
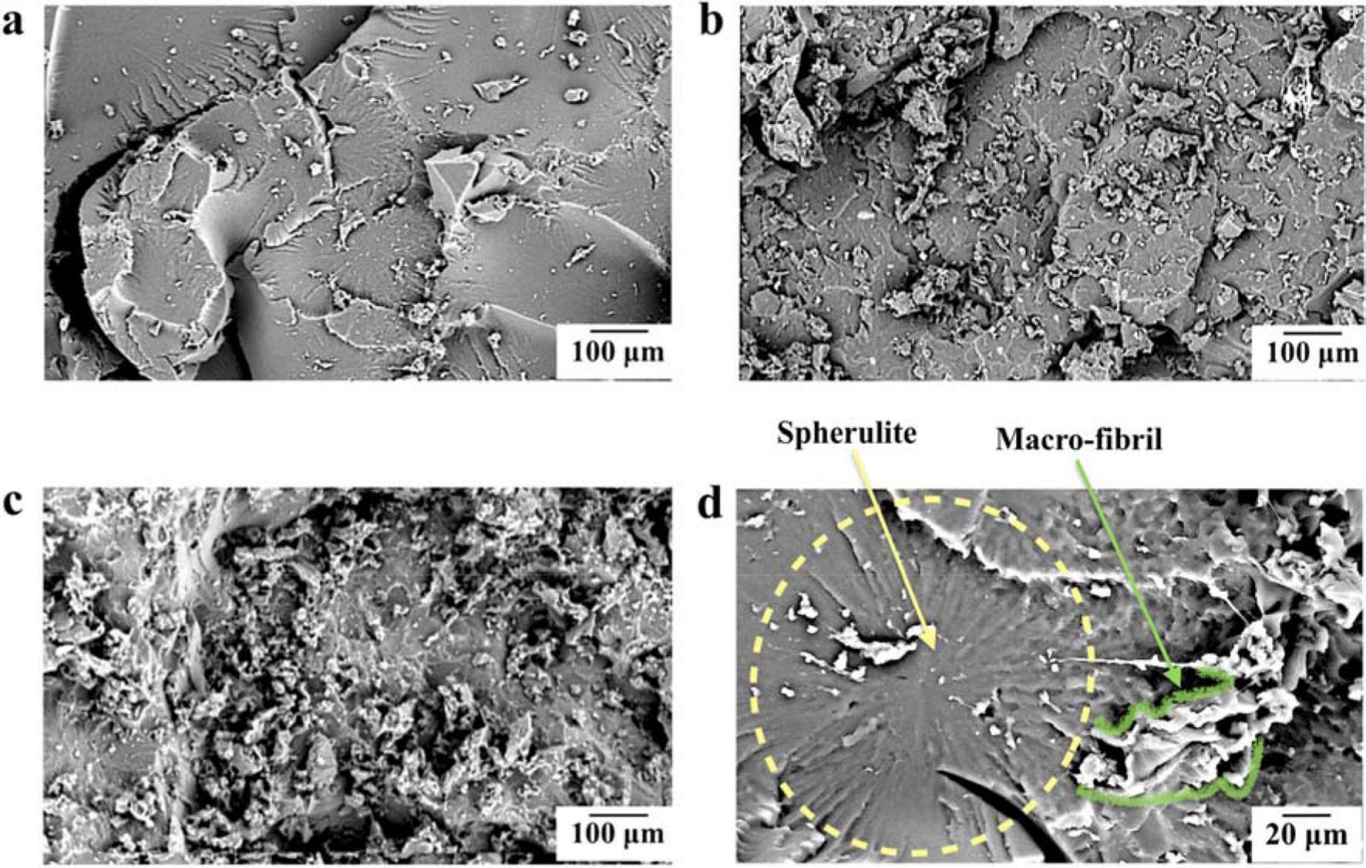
SEM fractographies of spalled surface: **(a)** neat PU; **(b)** nanocomposite without silane terminations; **(c)** HNT-PU nanocomposite. In **(d)**, we show the neat PU again at a different magnification, with a spherulite and macro-fibril highlighted. **(a)** is reproduced from^27^ under license CC BY 2.0.

From the spall plane fractographies (Fig. 2) of the recovered spalled samples it is possible to qualitatively identify a rougher fractured surface for the nanocomposites, with extensively plastic deformed regions. The progressively rougher fracture surfaces shown as one goes from Fig. 2a–c corresponds to progressively greater spall strength of the samples, going from the low strength neat PU in (a), the moderate strength nanocomposite without silane terminations in (b) and the strongest sample, the partially silane terminated nanocomposite, in (c). Greater roughness of the fracture morphology is evidence of a more energy dissipative failure mechanism. Spherulitic structures (quasi-spherical semicrystalline regions) can be seen clearly in the neat PU, and the visually flat fracture surface indicates a brittle fracture character of the spherulites.

Considering the domain segmented microstructure of PUs, we expect that the crystalline spherulitic structures are dispersed in a SS matrix^6^. Under the tensile strain rate magnitude of 10^4^ s^−1^ experienced by the samples during the spall testing, the PU tends to present a glassy-like behaviour and to fail in a brittle manner^32^. Based on the fractography results, the higher rigidity of the spherulitic structure would lead to flat fracture, while the more ductile interfacial regions would be able to sustain deformation. The interfacial regions are mainly filled with an amorphous composition that was pushed out during the crystallization^33^. These amorphous interspherulitic areas present PU macromolecules that are predominantly constituted by soft segments^34^. As such, the deformation of these amorphous regions would delay the spallation failure by resisting the applied stress though elongation, and the fracture of highly deformed regions would eventually lead to spall plane formation. For brevity, we will refer to these highly deformed regions as macro-fibrils, acknowledging their morphological similarity to smaller fibrils commonly seen in the deformation of polymers. Figure 2d presents the flat fracture surface of the spherulite and a macro-fibril at the spalled surface of the neat PU. During the spallation event the macro-fibrils’ response to the applied tensile stress increases the capacity of the polymer to plastically deform, and greater the density of macro-fibrils formed, the greater the fracture toughness. This observation is consistent with the progressively tougher response of the samples discussed earlier.

**Figure 3:**
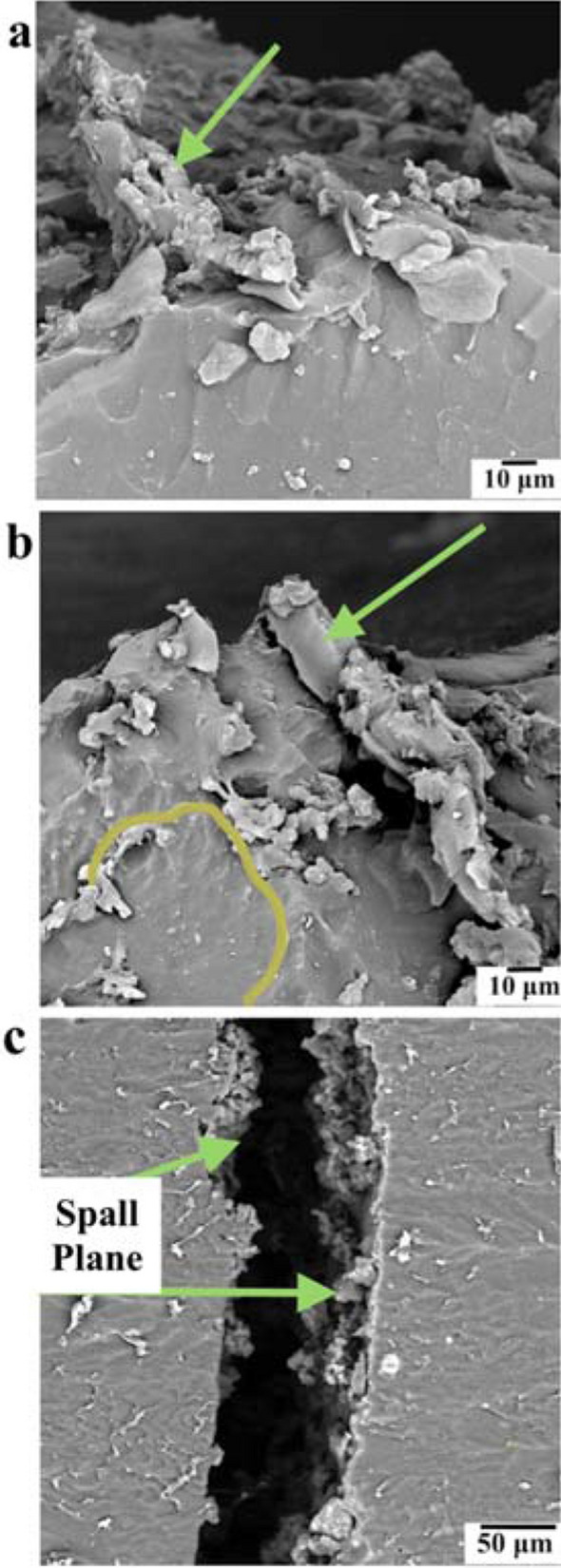
SEM fractographies of the neat PU spalled surface **(a)** side view with a macro-fibril indicated; **(b)** top view pointing out a macro-fibril and approximately indicating a spherulite; **(c)** side view of the spalled specimen.

In this manner, the more energy dissipative fractured surface of the nanocomposite can be associated with the presence of smaller and more rigid spherulites, which confer a higher density of macro-fibrils formed after the brittle failure of the spherulitic structure. Analysis of the spherulite size showed that the nanocomposite presented an average spherulitic diameter of 182 ± 12 µm, whilst it was 274 ± 13 µm for the neat PU. Figure 3a shows a side view fractography of the neat PU’s spalled surface, highlighting the presence of a deformed macro-fibril in the longitudinal direction. Figure 3b reveals the top view of the spalled surface, showing the presence of a flatter fracture in the surface of spherulitic structure near the macro-fibril. Finally, a lower magnitude side view of the spalled samples (Fig. 3c) displays the roughness of the resultant spalled plane.

**Figure 4:**
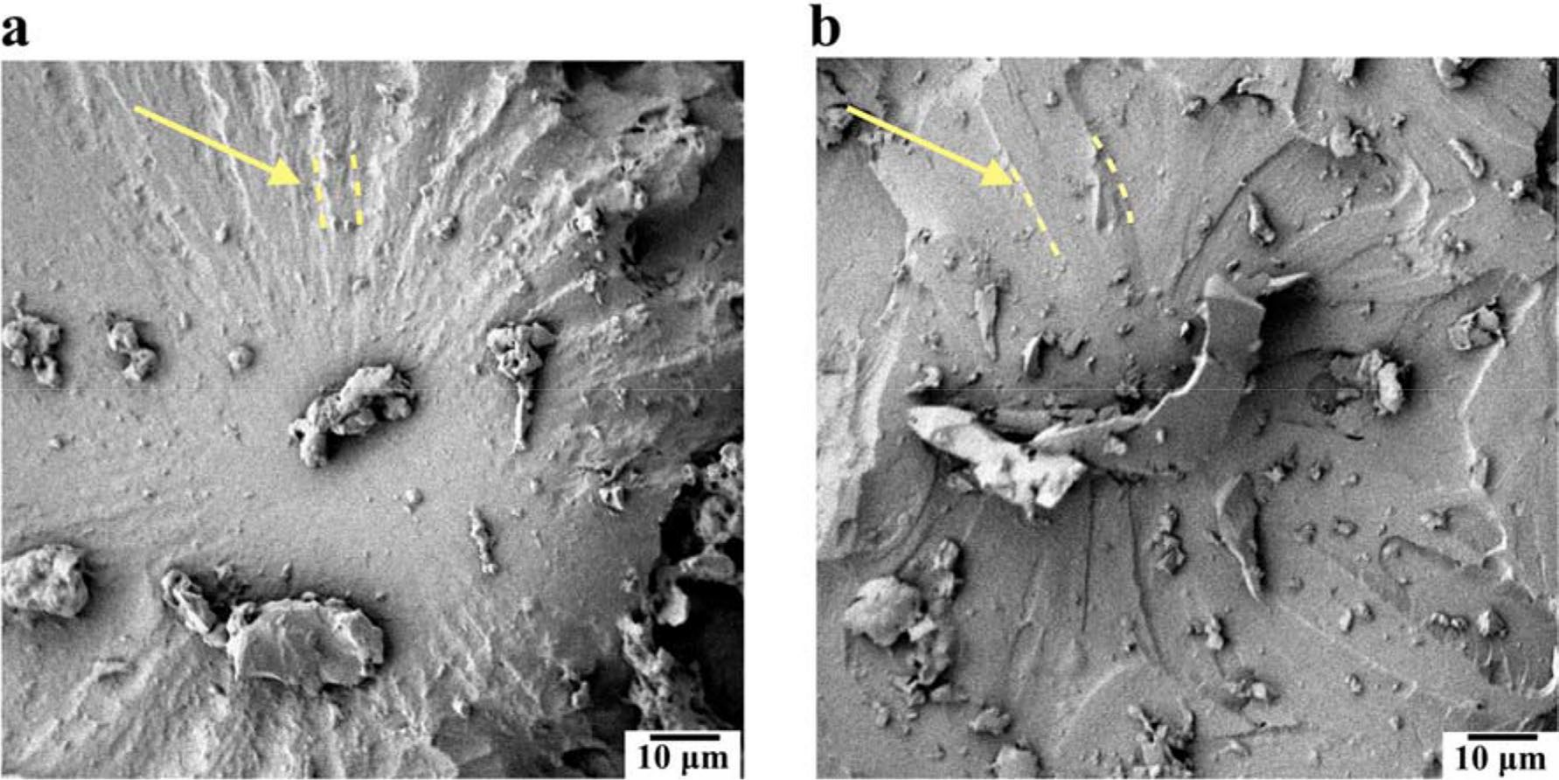
FESEM images of spherulitic texture in the **(a)** neat PU and **(b)** nanocomposite spalled surfaces with the discernible difference of thickness between lamellar crystal bundles indicated by dashed lines and arrows.

FESEM images (Fig. 4a,b) present the spherulitic structures of neat PU and nanocomposite. The lamellar crystal bundles can be identified radiating from the center of the spherulites^35^. Through the comparison of the spherulitic texture morphologies, we can see the presence of thicker lamellar crystal bundles for the nanocomposite. The difference in thickness of the lamellar crystal bundles is highlighted by arrows in Fig. 4. The morphology and crystallization kinetics of the spherulites are directly dependent on the thermal history experienced by the PU^36^. However, the neat PU and nanocomposite samples were submitted to the same thermal conditions, and yet a clear distinction between the lamellar morphologies is observed on the FESEM images. Considering that the content of hard segments within the nanocomposite was not significantly different than that in the neat.

PU (approximately 32 wt.%), and that the bonding of the HNTs to the hard segments has been shown to promote the micro-phase separation of the nanocomposite^37^, we expect the occurrence of spherulites with a higher concentration of crystallized hard segments in the nanocomposite. Indeed, DSC results from our previous study^27^ showed that the melting point of the hard phase crystallites was approximately 15 °C higher in the nanocomposite compared to that of the neat PU.

**Figure 5:**
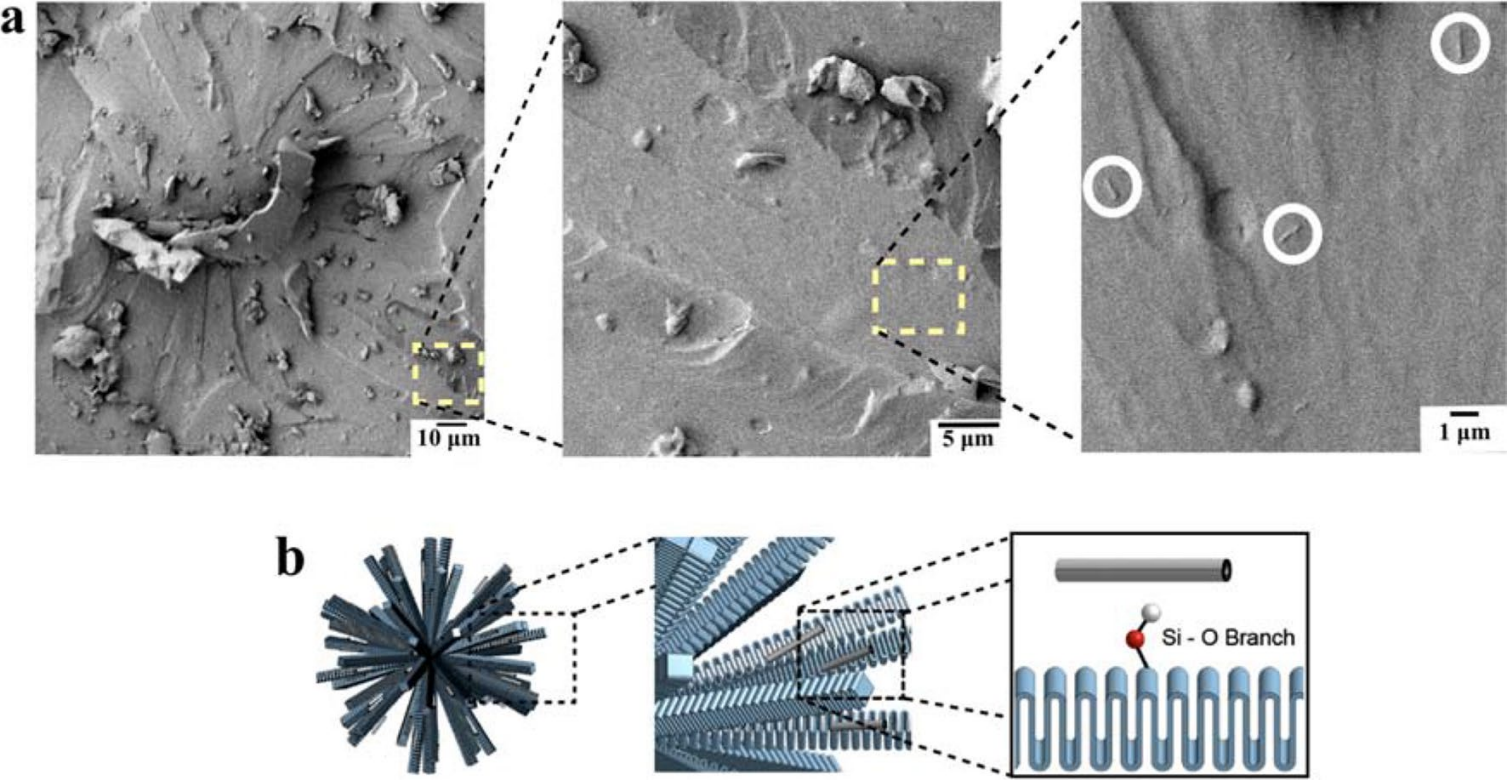
**(a)** FESEM images of spherulitic texture in the nanocomposite showing evidence of HNT positioning in the lamellar crystal bundle (circled in white) and **(b)** schematic representation of the spherulitic structure in the nanocomposite showing the bonding of hard segment chains with silane terminations to HNTs. **(b)** Created by Anton Lebar and used with permission.

The morphological structure of spherulites in thermoplastic PUs is such that the hard segment chains are arranged perpendicularly to the longitudinal direction of the lamellar crystal bundle structure^15^. The HNT nanotubes would likely be found in these lamellar crystal bundles, due to the bonding between the outer of surface hydroxyl groups of the HNT and the silanol groups in the silane terminations of the PU. Figure 5a presents evidence of HNTs embedded in the lamellar crystal bundles of the nanocomposite spherulitic structures. Figure 5b shows a graphical representation of the spherulites in the nanocomposite. The micrographs suggests a positive influence of the HNTs on the hard phase crystallinity. As observed by Maiz et al.^38^, the addition of efficient nucleation agents in PU can lead to an increase in the crystallinity, the lamellar thickness, the crystallization kinetics, and the crystallization and melting temperatures of the PU. Pourmohammadi-Mahunaki et al.^39^ have reported an increase in the crystallization and melting temperature of the hard phase, and a higher crystallization rate for the first phase of crystallinity with the addition of HNTs in PU, showing the intrinsic nucleation effect of the HNTs on the crystallinity of the PU.

It is worth emphasizing that the spall failure of traditional fiber reinforced polymeric materials usually shows a decrease of the spall strength compared to the neat polymer, due to increased void nucleation at the weakly bonded filler/polymer interface during spallation^40^. Huneault et al.^41^ reported the occurrence of carbon nanotube pullout at the spalled surfaces of epoxy reinforced with 1% wt. of carbon nanotubes. Their findings indicate that due to the weak adhesion at the filler/polymer interface, these regions would eventually act as nucleation sites for fracture resulting in a significant decrease in the spall strength. Lebar et al.^42,43^ studied the filler/polymer adhesion interference on the spall strength of polydimethylsiloxane and polyurethane reinforced with alumina particles. Their spall testing results were obtained for composites reinforced with pristine alumina (untreated), and for composites based on silane treated alumina with reduced interfacial chemical adhesion to the polymeric matrix. Their results point out that the reduction in the filler/polymer adhesion generates a significant decrease on the composite spall strength.

However, our results do not only show evidence of a good filler/polymer adhesion, they also indicate the ability of the HNT to be integrated into the final polymer microstructure. As shown in the FESEM images (Figs. 4b and 5a) we did not observe evidence of nanotube pullout, and the occurrence of different spherulitic morphologies suggests that the role of the HNT is to alter the final macromolecular organization of PU. This implies that HNTs in this system play a different role than a conventional reinforcement filler. Conventionally, the positive effects attributed to filler particles in a polymer are the action of the filler as a barrier to fracture growth via crack-front pinning and bowing, and fiber pull-out^44,45^. Here, we see that the HNTs affected the spherulitic structure development and therefore the final morphology of the polymer.

The failure mechanism of the spherulitic structure observed under high strain conditions differs from what is expected under quasi-static conditions for PU. In a quasi-static tensile testing configuration, one would expect the elongation of the soft domain matrix combined with the tilt of the hard segment lamellar domains. The spherulite structure would be deformed to an ellipsoidal shape, and upon stress release the spherical shape would be restored by retraction. Thus, the soft matrix presents an elastomeric behaviour with the hard domains acting as physical cross-link sites^7^. Under dynamic tensile loading, we have observed the brittle failure of the spherulites combined with the stretching of the interspherulitic regions. Similar to the response under quasi-static conditions, the ductility of the PU under dynamic conditions can be attributed to the soft domain stretching. However, the brittle fracture surface of the spherulites suggests that the dynamic failure of the hard segment lamellar domains is not accompanied by significant conformational motion.

**Figure 6:**
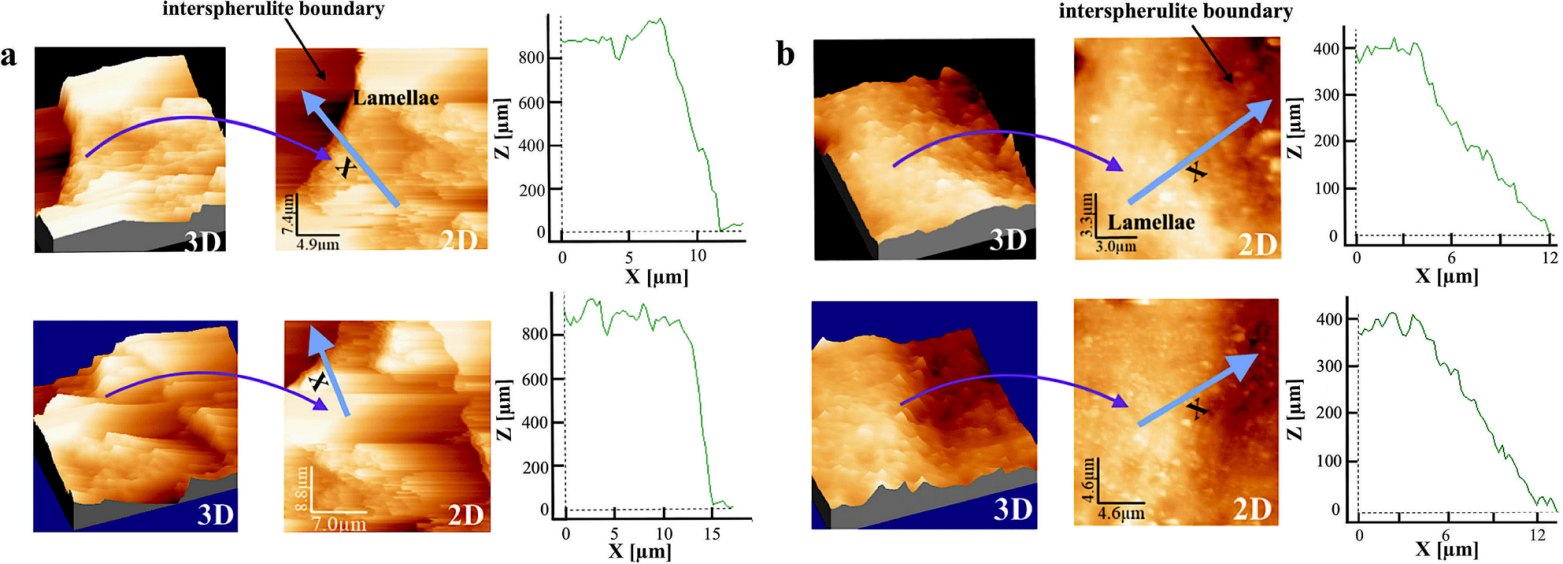
AFM height images and height profiles starting from the spherulite and ending at the interspherulitic regions (as presented in the 2D view) of **(a)** neat PU, and **(b)** nanocomposite.

The AFM topology images of the spherulite/interspherulitic interface of the unfractured samples is presented in Fig. 6. Comparing the height profiles starting from the lamellar crystal bundle and finishing in the amorphous interspherulitic matrix for both materials, the height profile for the neat PU displays a more abrupt transition between the spherulitic and amorphous regions. The vertical height difference (∆z) between hill and valley is around 900 nm for the neat PU, and 400 nm for the nanocomposite. This difference in the height profile may be related to a higher volumetric contraction of the coarse spherulitic structure in the neat PU during crystallization. This would consequently increase the void formation tendency along the boundaries and result in weaker and more brittle boundaries in the final microstructure^46^.

**Figure 7:**
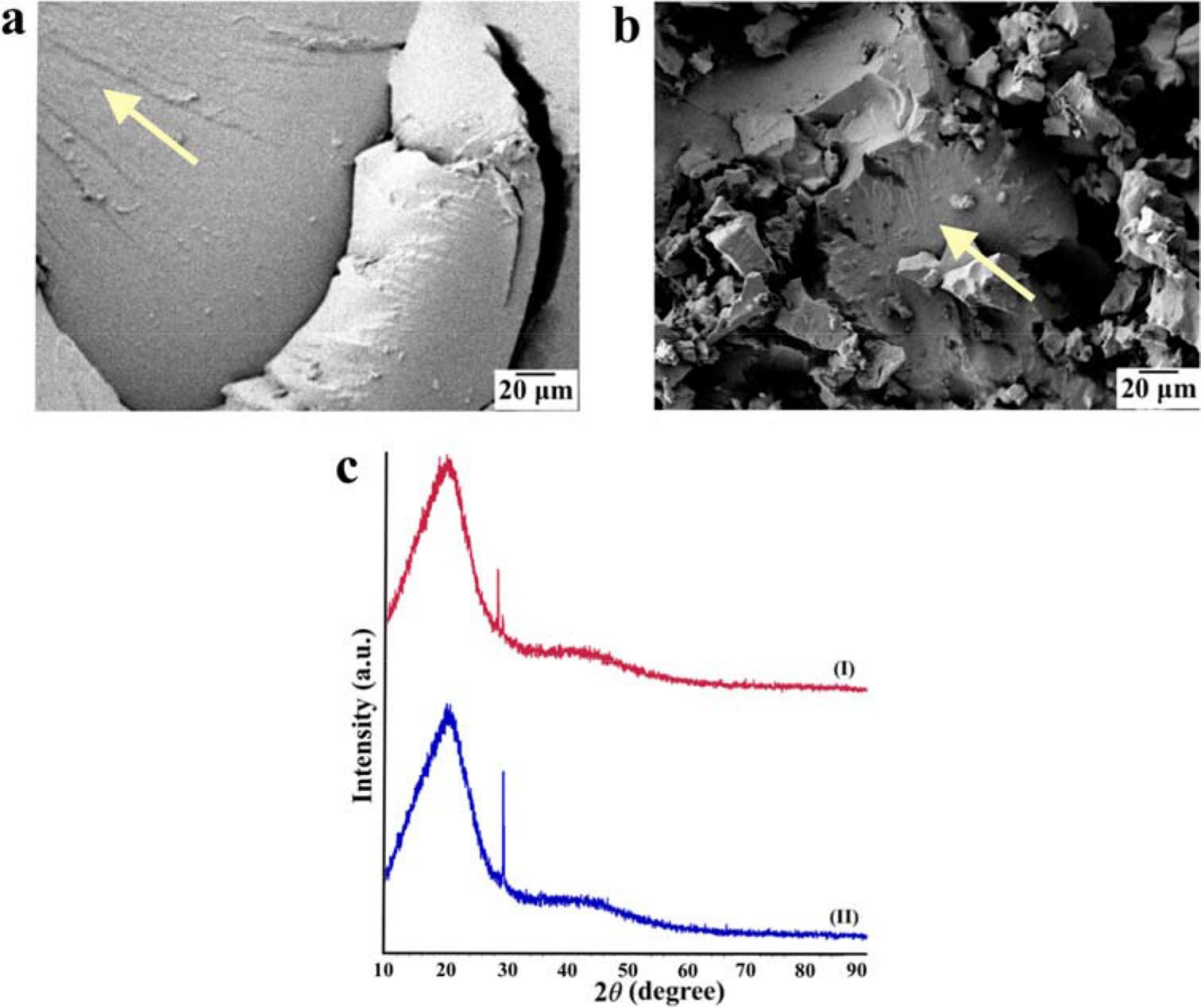
**(a)** Fractographies showing interspherulitic damage (the spherulites can be seen highlighted by arrows) for the neat PU and **(b)** nanocomposite; **(c)** X-ray diffractograms of the neat PU (I) and nanocomposite (II).

Furthermore, the bonding of the HNTs to the hard segments promotes the micro-phase separation of the nanocomposite, which results in a higher chain mobility of the soft segments^37^. As the interspherulitic regions of the PU are mainly formed by soft segments, the presence of a less restricted molecular motion can lead to a more ductile behaviour of these regions. Thus, the higher volume contraction, combined with a higher hard segment concentration in the interspherulitic regions, are factors that contribute to the more brittle behaviour experienced by the neat PU. In the fractography images (Fig. 7a,b) it is possible to observe a more brittle behaviour in the interspherulitic regions of the neat PU, and for the nanocomposite a more ductile behaviour with extensively deformed regions around the spherulite.

The crystallinity of the samples was studied via XRD (Fig. 7c). The neat PU and nanocomposite presented two broad diffraction peaks at approximately 20° and 45°, which can be attributed to soft segments in the PU^47^. These peaks present similar intensities on both samples but are slightly sharper for the nanocomposite, which may suggests a higher crystal regularity for the nanocomposite^48^. The peak at 29.5° was substantially higher for the nanocomposite, indicating the occurrence of more ordered crystallites, and the shift from 28.6° (neat) to 29.5° (nanocomposite) indicates a more tightly packed crystal structure in the nanocomposite. This is all evidence supporting the notion that the HNTs interact with the hard domains to help create a more ordered and stronger spherulitic structure. Finally, we note that the introduction of a low content of HNT did not introduce new peaks in the X-ray diffractograms, suggesting a good dispersion of the nanotubes^49^.

## Conclusion

The higher values of fracture toughness and dynamic tensile strength of the nanocomposite can be mainly attributed to the following factors.

### Finer, more rigid spherulites in the nanocomposite

The presence of smaller and more rigid spherulitic structure in the nanocomposite led to a higher fracture energy during the spallation process. The FESEM images showed the thicker lamellar crystal bundle structure of the nanocomposite with dispersed HNTs, while XRD results presented evidence of a more ordered and packed crystal structure, indicating a positive effect of the nanotubes on the hard phase crystallization. We believe that further studies investigating the influence of the HNTs in the nucleation and growth processes of the spherulitic structure are warranted.

### Brittle interspherulitic regions in the neat PU

The neat PU fractographies present coarse spherulites with some voids and cracks propagating in the interspherulitic regions. Considering the macro-fibril stretching of the soft segment matrix during tensile loading, the presence of brittle interspherulitic interfaces would accelerate the spall fracture. AFM height profiles indicate a greater height transition between the spherulite and interspherulitic regions for the neat PU, which could be related to a higher volume contraction of the coarse spherulites in the neat PU during crystallization. These results reinforce the possibility that the neat PU present weaker spherulitic boundaries, as the increase in the volume contraction can increase the possibility for void nucleation to occur along the interspherulitic regions. Also, taking into consideration the lower degree of micro-phase separation in the neat PU compared to the nanocomposite, the higher concentration of hard segments in the interspherulitic regions of the neat PU can interfere with the mobility of the soft segments and reduce the overall ductility of these regions.

### Higher interspherulitic area in the nanocomposite

A higher density of macro-fibrils is formed for the nanocomposite, as can be seen in the fractographies. The presence of finer spherulites confers to the nanocomposite a higher density of interspherulitic boundaries. Taking into account that these regions are mainly constituted by a soft segment matrix and that these areas sustain the highest degree of plastic deformation during the spall plane formation, it follows that as these macro-fibrils are more distributed, and a more energy-dissipative, tough response will result.

## Methods

### Materials

The PU prepolymer (Poly (propylene glycol), tolylene 2,4) was purchased from Taiwan PU corporation and (3-(N-ethylamino) isobutyl) trimethoxysilane obtained from Gelest Inc. The curative (4,4’-Methylenebis 2-chloroaniline) and HNT nanotubes, having diameters in the range of 30–70 nm, lengths of 1–3 μm, and surface area of 64 m2/g were supplied by Sigma Aldrich. The hard segment contents were calculated based on the weight fractions of diisocyanate and chain extender in the PU.

### Sample preparation

During the synthesis of the nanocomposites, the HNTs were added into the liquid pre-polymer at a weight fraction of 0.8% and were dispersed via a sonication process. Next, the partial silane termination occurred for an aminosilane content equivalent to weight fraction of 0.6% of the pre-polymer weight. As a result of the partial termination process, monodentate urea linkages and trimethoxysilane terminations were created in the PU’s prepolymer^50^. A small content of aminosilane was adopted to inhibit a substantial increase in the viscosity of the HNT/prepolymer solution. Additionally, the same curative was chosen for the neat PU and nanocomposite. The neat PU and nanocomposites were annealed for 22 h at 120 °C in a metallic mold. During the post-curing stage, the hydrolysis of the silane terminations can potentially produce silanol terminations in the prepolymer end groups, which can possibly react via condensation with surface hydroxyl groups of HNT^26^. Thus, the adhesion between the PU and nanotubes is promoted. The detailed synthesis and post-curing process can be found in our previous work^27^.

### Spall testing

The testing was performed in a 64-mm smooth-bore single-stage light gas gun at the Impact Research Lab facility at Carleton University. Acrylic flyer plates (3.2 mm thick) were used to induce spall in the neat PU and nanocomposite, according to the plate rigidity and shock Hugoniot similarity requirements for plate impact configurations^41^. The resultant strain rate in the samples were controlled by varying target specimen thicknesses. The back-face velocity histories of the samples were measured with a two-channel photonic Doppler velocimeter (PDV)^51,52^. A summary of the experimental results can be seen in Table 1. The acoustic properties of the polymers were determined using an Olympus 45MG ultrasonic thickness gage coupled with a delay line transducer at a frequency of 10 MHz. The sound speeds were measured to be 1.98 km/s and 2.03 km/s for the neat PU and nanocomposite, respectively.

**Table 1:** Summary of the experimental spall results.

Material	ε_u_ (10^4^ s^−1^)	σ_sp_ (MPa)	Shock stress (GPa)
Neat PU	2.75	105 ± 2	0.78
Neat PU	3.03	110 ± 2	0.94
Neat PU	3.16	134 ± 4	0.96
HNT-PU	2.76	143 ± 3	0.93
HNT-PU	2.83	149 ± 2	0.99
HNT-PU	2.81	151 ± 7	0.67
HNT-PU*	2.79	129 ± 3	0.93

### SEM and FESEM analysis

SEM fractography analysis of the recovered samples after the spall tests were recorded using a Tescan Vegal microscope. The HNTs were identified by using INCA Energy-dispersive X-ray spectroscopy analysis together with SEM. The morphology of the spherulitic structure was analyzed via FESEM imaging of the spall plane surface using a JSM-7500F FESEM (JEOL). The samples used for the SEM and FESEM were sputter coated with a 10 nm thick layer of gold. The average spherulitic diameters were found by using an image analysis program Fiji (ImageJ) based on the measurement of 30 spherulites for each material^53^. The standard deviations of the measurements were 12 μm for the neat PU and 13 μm for the nanocomposite.

### AFM analysis

Top surfaces (the sides of the sample open to air during polymerization and annealing) of neat PU and nanocomposites specimens were analyzed via AFM (Dimension 3100, Veeco Corp. Santa Barbara, CA, USA) in tapping mode with an aluminum-coated silicon tip (f_0_ = 278 kHz, r < 10 nm) installed. The largest scan range of the instrument was 100 μm × 100 μm. The surface topology of the interface between the spherulitic structure and the interspherulitic region was analyzed using the height images and surface profiles^54^. The AFM results analysis was performed using the WSxM software^55^.

### XRD examination

The analysis was performed with a Rigaku Ultima IV Diffractometer (CuKα source with λ = 1.54184 Å) with a scintillation counter and diffracted beam monochromator, using a scanning range (2θ) of 10°–90° with a 0.02° step size at a scan rate of 0.5°/min.
